# Animal models for bone tissue engineering and modelling disease

**DOI:** 10.1242/dmm.033084

**Published:** 2018-04-23

**Authors:** Jacqui Anne McGovern, Michelle Griffin, Dietmar Werner Hutmacher

**Affiliations:** 1 Centre in Regenerative Medicine, Institute of Health and Biomedical Innovation, Queensland University of Technology, Brisbane 4059, Australia; 2 Charles Wolfson Center for Reconstructive Surgery, Royal Free Hospital, London, NW3 2QG, UK; 3 UCL Centre for Nanotechnology and Regenerative Medicine, Division of Surgery and Interventional Science, University College London, London, WC1E 6BT, UK; 4 George W Woodruff School of Mechanical Engineering, Georgia Institute of Technology, Atlanta, GA 30332, USA; 5 Institute for Advanced Study, Technical University Munich, Garching 85748, Germany

**Keywords:** Bone regeneration, Bone defect, Bone metastasis, Tibia segmental defect, Cancer xenograft, Scaffolds, 3D printing, BMPs

## Abstract

Tissue engineering and its clinical application, regenerative medicine, are instructing multiple approaches to aid in replacing bone loss after defects caused by trauma or cancer. In such cases, bone formation can be guided by engineered biodegradable and nonbiodegradable scaffolds with clearly defined architectural and mechanical properties informed by evidence-based research. With the ever-increasing expansion of bone tissue engineering and the pioneering research conducted to date, preclinical models are becoming a necessity to allow the engineered products to be translated to the clinic. In addition to creating smart bone scaffolds to mitigate bone loss, the field of tissue engineering and regenerative medicine is exploring methods to treat primary and secondary bone malignancies by creating models that mimic the clinical disease manifestation. This Review gives an overview of the preclinical testing in animal models used to evaluate bone regeneration concepts. Immunosuppressed rodent models have shown to be successful in mimicking bone malignancy via the implantation of human-derived cancer cells, whereas large animal models, including pigs, sheep and goats, are being used to provide an insight into bone formation and the effectiveness of scaffolds in induced tibial or femoral defects, providing clinically relevant similarity to human cases. Despite the recent progress, the successful translation of bone regeneration concepts from the bench to the bedside is rooted in the efforts of different research groups to standardise and validate the preclinical models for bone tissue engineering approaches.

## Abstract (precis)

**Summary:** This Review describes the currently used animal models of critical-sized bone defects and the regenerative bone tissue engineering strategies used in these models, as well as the application of bone tissue engineering for the advancement of cancer xenograft models.

## Introduction

**Fig. 1.**
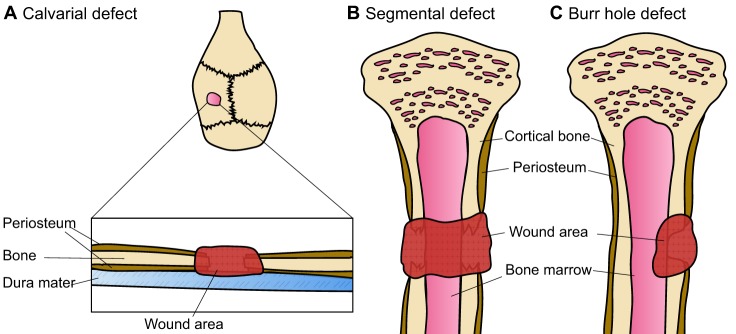
**Prevalent bone defect models.** (A) Calvarial defects are generally created via the introduction of a circular burr hole and the subsequent removal of the resulting bone disk. The surgery is performed in a manner so as to not damage the underlying dura mater. (B) In the segmental bone defect model, a larger and completely penetrating bone defect is generated. A segment of the bone is surgically removed, leaving a large and non-joining wound area (gap) between the bone edges. The gap is usually stabilised with a fixation device and/or filled with a tissue-engineered bone substitute to stimulate bone healing and to study bone formation. (C) In the burr hole, or partial defect model, an incomplete hole is drilled into the side of the bone to create a wounded area. The burr hole usually penetrates the cortical bone and can extend into the underlying cancellous bone or the bone marrow cavity. In this model, usually only one side of the bone is wounded.

The bone is a multifaceted organ consisting of several tissues, such as the cortical bone (see Glossary, Box 1), the cancellous bone with the marrow compartment and the periosteum (Box 1, Fig. 1). Each tissue is composed of a number of differentiated and precursor cells. While most bones in the body arise from the mesoderm (Box 1) during embryonic development, the calvarial bone arises from both the neural crest (Box 1) and the mesoderm layers (Couly et al., 1993). Together, these diverse bone constitutions form a complex organ with key physiological roles within the body. The biomechanical properties of the skeletal system play a crucial role in structural support, load-bearing for movement and physical protection of the inner organs. Furthermore, in addition to mineral storage and calcium homeostasis roles, the bone organ has important haematopoietic (Box 1) and immunological functions as the site of blood cell generation and immune cell differentiation. Significant bone loss can occur due to trauma or disease, such as cancer (Bauer and Muschler, 2000; Hollinger and Kleinschmidt, 1990). Therefore, bone engineering techniques present an avenue of research with the goal to regenerate the lost bone and restore its function.

**Box 1. Glossary**

**Calvarial bone(s):** The flat and compact bones of the skullcap, consisting of the frontal, parietal and occipital bones

**Cell homing:** A process in which cells migrate, or are recruited to populate, a new niche or location in the body following mobilisation from their site or origin

**Clonality:** The result of proliferation as determined by the cell of origin, in which the daughter cells remain phenotypically and genetically identical to the parent cell

**Cortical bone:** The dense and compact outer surface of the bone organ that protects its inner cavity

**Corticoperiosteal flap:** A vascularised tissue flap from near the region of the knee that can be used to vascularise a critical-sized bone defect

**Critical-sized defect (CSD):** A wound in a particular bone that will not spontaneously heal during the organism’s lifetime; or that will not reach greater than 10% bone regeneration within the duration of the experimental time course

**Ectopic:** Something occurring outside its normal place or position

**Erythroluekaemia:** A relatively rare malignancy of the haematopoietic system resulting in proliferation of the erythroid and myeloid bone marrow progenitor cells

**Femoral neck:** The region of the thigh bone connecting the femur to the femoral head

**Femur:** The thigh bone of the leg

**Growth plates:** The region in a long bone where longitudinal growth occurs

**Haematopoietic:** Of blood-forming capacity

**Histocompatible:** Cells or tissues with antigenic similarity between donor and recipient and therefore do not illicit an immune rejection response

**Mesoderm:** One of the three primary germ layers of early embryos. The mesoderm is the middle layer, between the ectoderm (outer layer) and the endoderm (inner layer), and goes on to form connective tissue and muscle during development

**Metatarsus:** The group of five long bones that comprise the foot, between the hind-foot and toes

**Neural crest:** A group of cells arising from the early embryonic ectoderm layer which gives rise to cells such as melanocytes, craniofacial cartilage and bone, smooth muscle, peripheral and enteric neurones as well as glia

**Osteoconductive properties:** Properties that promote the attachment of bone-forming cells (mesenchymal progenitor and pre-osteoblastic cells); for example, a scaffold or matrix for bone repair

**Osteoinductive factors:** Factors which induce bone-forming cells to become bone

**Osteoprogenitor:** A mesenchymal cell which can differentiate into an osteoblast

**Orthotopic:** Something which occurs at its normal place and position

**Periosteum:** The membrane of dense and vascular connective tissue covering the outer surface of all bones, excluding the joints

**Sagittal incision:** A cut from top to bottom of an anatomical structure, diving the structure into left and right portions

**Secondary osteonal remodelling:** A process of bone remodelling where the primary bone (present since fetal development) is replaced with secondary bone, which has a lower density of osteocytes

**Tibia:** The shin bone of the leg

**Tricalcium phosphate:** A biodegradable calcium salt of phosphoric acid [chemical formula Ca_3_(PO_4_)_2_]. Available in α-, α′- and β-polymorphs, with β-tricalcium phosphate having the highest crystallographic density.

**WNT protein signalling:** Cell signal transduction pathway that uses proteins to pass signals into the cell through cell surface receptors. Has a role in carcinogenesis, embryonic development and tissue regeneration

In addition to its role in providing structural rigidity and support to the body, the bone organ also contains bone marrow, which plays an important role in stem cell maintenance and gives rise to all the cellular components of the blood (Mendelson and Frenette, 2014). Haematopoiesis occurs mainly in the specialised bone marrow microenvironment, which houses the haematopoietic stem cell niches. Other cells within the bone marrow include osteoblasts, osteocytes and osteoclasts, in addition to bone marrow-derived mesenchymal stem cells (BMSCs), adipocytes and vascular endothelial cells (Mendelson and Frenette, 2014). Importantly, the haematopoietic niche of the bone marrow has been reported to play an important role in the development and potentiation of primary and secondary bone malignancies.

Given the important functional roles of the bone, regeneration of the bone organ following trauma or surgery to resect a malignancy is crucial to re-establish form and function in the body. Despite the remarkable innate regenerative capacity of the bone, repair of large defects is still clinically challenging. The current gold standard for restoring bone defects is autologous grafting, as it is histocompatible (Box 1) and nonimmunogenic, but this is not without the risk of donor-site morbidity and limited tissue availability (Bauer and Muschler, 2000). In addition, allogenic grafts and demineralised bone matrices have been used to treat bone defects, but pose a risk for immune-mediated rejection and transmission of disease (Oakes et al., 2003). Although these clinically established methods improve outcomes for patients with bone defects, they could be improved upon in terms of safety, cost and the ability to restore large defects.

The application of tissue engineering strategies using specialised constructs to repair bone defects has many advantages over the current bone grafting techniques, as the newly formed bone will be regenerated from the patient's own cells and will fully integrate with their existing skeletal system. Tissue-engineered constructs (TECs) are generally composed of a biocompatible scaffold that can replicate the native bone structure, osteogenic cells such as osteoblasts or BMSCs that can populate the scaffold and form new bone, and growth factors such as bone morphogenetic proteins (BMPs) that send signals to the cells in the local area to produce new mineralised bone matrix and vascularise the site (Hartman et al., 2005; Hofmann et al., 2013; Kirby et al., 2016). These scaffold, cell and growth factor components of the TEC work synergistically to stimulate bone formation at the site of implantation.

Bone formation in a TEC largely relies upon the construct's integration with the host vasculature in order to supply oxygen and nutrients, and to allow for host cell homing (Box 1). Furthermore, bone formation is highly dependent on the physicochemical and biochemical properties of the TEC, such as the presence of osteogenic cell sources to create new bone tissue, the availability of BMPs and other osteoinductive factors (Box 1) which commit local cells to osteoblastic differentiation, and the osteoconductive properties (Box 1) intrinsic to the construct itself to support the formation of new bone (Fröhlich et al., 2008; Muschler et al., 2010). While larger animal models such as sheep and pigs are used to study tissue-engineered (TE) bone for the regeneration and repair of critical-sized defects (CSDs; Box 1), mouse models are preferred for ectopic (subcutaneous; Box 1) TE bone formation studies. Mice are favourable in this context, owing to the availability of highly immunocompromised strains which are permissible to the engraftment of human tissues. Unfortunately, due to their size and the surgical challenges this presents, mouse models are not commonly used for studying orthotopic (Box 1) integration of TE bone.

The ability to generate large-volume and functional TE bone is critical for clinical translation owing to the load-bearing nature of the tissue. Based on the physical properties of the bone, many TE studies assess the resulting effects via measurements of bone volume and mineralised tissue formation and density using techniques such as X-ray or computed tomography (CT) analysis (Martine et al., 2017; Nakamura et al., 2017). Histological techniques are also commonly employed to investigate mineralised tissue formation, integration with the host, identification of cellular components such as marrow and vasculature, and the host inflammatory response to the implanted TEC (Hofmann et al., 2013; Reichert et al., 2010b; Scotti et al., 2013). Together, these techniques can provide a comprehensive assessment of the engineered bone and its effectiveness in mitigating bone loss.

Traditional bone regeneration techniques using TEC are now being applied to disease modelling, advancing xenograft studies for improved cancer research. Methods to humanise the bone and bone marrow for implantation in immunocompromised mouse models have gained momentum as an avenue to study primary and secondary bone malignancies (Martine et al., 2017; Reinisch et al., 2016). Bone tissue engineering and regenerative medicine (TE&RM) techniques can also be utilised to study the complex bone organ in both normal and disease states. Recently, several *in vivo* models have been developed which use TE approaches with the aim of mimicking the physiological conditions of a functionally intact organ bone, ‘humanising’ mice to generate as much human-like tissue as possible within the murine host in order to study the species-specific mechanisms of human malignancies (Holzapfel et al., 2014; Moreau et al., 2007; Thibaudeau et al., 2014).

In this Review, we first provide an overview of the current traditional bone TE techniques and how they are used to study bone repair in animal models. Specifically, we discuss the commonly used *in vivo* bone defect models, the prevalent species in which these studies are conducted, as well as the TE techniques employed for bone regeneration and repair. Second, we discuss the developments made in rodent models that utilise TE bone to study bone-related malignancies. In this section, we describe the application advances of TE bone models and the current *in vivo* research avenues into primary bone cancers, such as osteosarcoma and leukaemia, as well as secondary bone malignancies, including breast and prostate cancer metastases.

## Models of bone defects

Bone defects are serious conditions in which a part of the bone is damaged or missing owing to trauma or surgery, and need to be repaired through interventional techniques such as bone grafting. There are many animal models being used to evaluate bone graft substitutes, but the main four types are the calvarial defect, long bone or segmental defect, partial cortical defect and cancellous bone defect models (Bigham-Sadegh and Oryan, 2015) (Fig. 1). The segmental and calvarial bone defects are the most widely described and used in the literature (Bigham-Sadegh and Oryan, 2015).

### Calvarial bone defects

The calvarial bone defect is usually carried out in rodent species. Rodents continue to remodel their skeleton throughout their lifetime, with the growth plates (Box 1) remaining open throughout adulthood (Gomes and Fernandes, 2011). The calvarial bone defect procedure is very simple. The rat calvarial defect involves creating a sagittal incision (Box 1) across the scalp of the animal. A flap is then raised to expose the calvarial bone and a standardised circular bone defect spanning the entire depth of the bone is created (usually the parietal bone; Fig. 1A) using a trephine bur with saline irrigation to prevent damage to the surrounding host bone. The excised bone disk is removed to prevent damage to the dura mater. The periosteum is then repositioned and the overlying skin flap is closed with sutures (Nakamura et al., 2017). Several groups have utilised the calvarial model to evaluate different TE scaffold types including synthetic and natural materials, with and without cells and growth factors such as BMPs (Table 1).

**Table 1.**
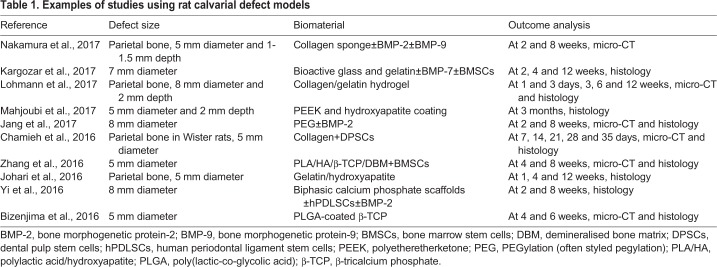
**Examples of studies using rat calvarial defect models**

#### Considerations when using the calvarial model

When generating any bone defect model, the size of the induced defect is of utmost importance, in particular the CSD (Reichert et al., 2009). In rats, there has been controversy about what the optimal dimensions of the CSD are (Bosch et al., 1998; Gomes and Fernandes, 2011; Hollinger and Kleinschmidt, 1990). To establish a CSD in the rat calvarium, defects that are 5 mm in diameter are most commonly used. Bosh et al. demonstrated that after 12 months, minimal bone formation was observed at the defect margins in rats (Bosch et al., 1998). The authors concluded that the advantages of this 5 mm defect are the ability to induce two defects per animal and avoidance of the sagittal suture spanning the defect. However, some advocate for 8 mm defects as CSDs for rats, due to Hollinger et al. demonstrating ∼10% *de novo* bone formation after 13 months in the 5 mm defect, thereby failing to meet the accepted criteria for classification as a CSD (Gosain et al., 2000; Hollinger and Kleinschmidt, 1990). However, generating the 8 mm defect requires a lateral-based craniotomy approach, which could impair the local regenerative process and impair overall bone healing (Gomes and Fernandes, 2011). In mice, 4 mm defects are considered CSDs, but both larger and smaller defects have been reported (Ducheyne et al., 2011). Furthermore, it is clear that the age and strain of the animal also determine the CSD in both the mouse and the rat (Gomes and Fernandes, 2011).

#### Advantages and disadvantages of calvarial models

The calvarial bone defect model is popular among researchers, as the bone structure allows for the generation of a standardised defect that can be analysed using histology and radiographic analysis (Gomes and Fernandes, 2011). Furthermore, biomaterials can be inserted with adequate surgical access without the need for external fixation owing to the support provided by the dura mater and the skin (Gomes and Fernandes, 2011). Moreover, rodent models are inexpensive, easy to house and evoke limited social concern (Gomes and Fernandes, 2011). Several biomaterials have been assessed using the calvarial model, allowing for good comparisons of the differences between different TE scaffolds. One drawback of the calvarial model is the inability to assess the performance of the TE biomaterial under physiological mechanical loads, which is important for some clinical applications of bone TE, such as the regeneration of load-bearing bones (Gomes and Fernandes, 2011). Furthermore, rodent models are not useful for long-term studies where multiple biopsies or blood samples are needed due to their short lifespan and relatively small tissue and blood volumes compared with those of humans and larger animal models such as sheep and pigs. Moreover, when it comes to clinical application, rodent models begin to fail to answer questions to ascertain the effectiveness of the TE strategy because of their differing skeletal loading patterns. To overcome these limitations, segmental bone defects in long bones of large animals can be used to more closely mimic the clinical scenario.

### Models of long-bone segmental defects

Large animal models have been developed to assess the effectiveness of tissue engineering strategies in situations that more closely mimic the clinical scenario. In the majority of reports, the CSD in long bones is created using an osteotomy approach whereby a drill or saw is used to remove the required segment from a predetermined site in the bone (Fig. 1B,C) (Berner et al., 2013; Cipitria et al., 2013, 2015; Reichert et al., 2010a). If the study requires modelling of a traumatic defect, then the edge may be left uneven.

Long-bone segmental defects have been modelled in several species, including dogs, sheep, goats and pigs (Reichert et al., 2009), and a number of factors should be considered when selecting an animal species for long-bone defect modelling studies. These involve evaluating the similarities of the species to the human physiology, technical operative ability, and animal availability and cost (Table 2) (Reichert et al., 2009).

**Table 2.**
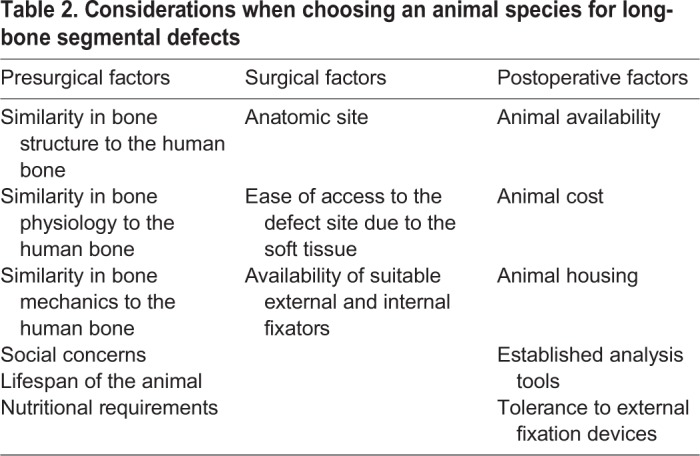
**Considerations when choosing an animal species for long-bone segmental defects**

The bone composition of the dog, sheep, goat and pig is similar to that of humans (Newman et al., 1995). Dogs were traditionally used as a model in orthopaedic research but, due to public concerns, their use in segmental defect models has decreased (O'Loughlin et al., 2008). Pigs have been used in bone regeneration research; however, the requirement for careful handling of the species often deters their use. Thus, the most commonly used animal species for segmental bone defects is the sheep (Table 3). Mature sheep have a similar body weight to adult humans, allowing for easy translation of the findings to the clinical setting (Reichert et al., 2009). Furthermore, the mechanical loading of ovine (sheep) hind limbs is well documented, and around half of that in humans during the walking phase (Taylor et al., 2006), further easing the translation of research findings. Moreover, sheep and humans have similar metabolic and bone remodelling rates (den Boer et al., 1999).

**Table 3.**
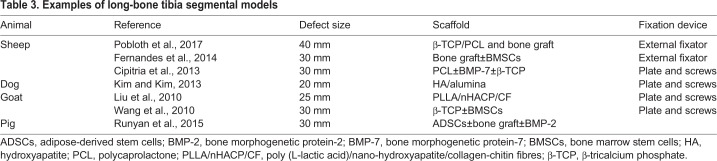
**Examples of long-bone tibia segmental models**

#### Long-bone tibia segmental defect

Similarly to the calvarial models, the size of the defect in long bones must be carefully considered to create a true CSD. The segmental defect is caused by removal of a length of bone by a drill or saw, and is usually performed clinically in response to trauma, or from resection of dead, infected or nonhealing bone (Fig. 1B). It is generally accepted that the size of the defect should be 2-2.5 times the diameter of the long bone's shaft (ASTM, 2014; Lindsey et al., 2006). However, a CSD is not only determined by its size, but also by other factors, such as the age and species of the animal, the defect location, bone structure, presence of the periosteum, mechanical loading of the bone and the metabolic and systemic condition of the animal, as well as by the fixation device used to stabilise the bone defect and to permit an early return to mobility (Glatt and Matthys, 2014).

Several long-bone sites have been used to demonstrate segmental bone defects, including the proximal third of the tibia, the femoral neck and metatarsus (Box 1) (Christou et al., 2014). The tibia is the most commonly used anatomical site in ovine models. Most studies in sheep report a CSD in the tibia to be 2-2.5 times the diameter of the bone (ASTM, 2014; Gugala et al., 2007; Lindsey et al., 2006), but there are reports of using three times the diameter (Gugala et al., 2007). However, many studies often only report the length of the defect and not the size of the bone itself, making it unclear whether the induced defect is truly a CSD (Gugala et al., 2007). Chirsou et al. also found that in a breed of sheep with an average midshaft diameter of 22 cm, a 50 mm diameter was sufficient to cause a CSD (Christou et al., 2014). Recently, Lammens et al. proposed the use of a large 4.5 cm defect with a polymethylmethacrylate (PMMA) spacer as more suitable than a 3 cm defect in the ovine tibia defect model (Lammens et al., 2017). Hence, the understanding and consensus of the CSD in long bones remains unclear (Gugala et al., 2007).

However, the CSD itself is not the only aspect of long-bone segmental defects that is being evaluated. Several studies have evaluated different biomaterials in the tibia defect model (Mastrogiacomo et al., 2006). Among different TE scaffold types, ceramic-based scaffolds have been favoured due to their osteoconductivity characteristics. Ceramic scaffolds composed of 100% calcium phosphate demonstrated progressive bone formation in a 48 mm ovine tibia defect over 1 year, with the scaffolds being completely resorbed by 2 years (Mastrogiacomo et al., 2006). The addition of stem cells to ceramic scaffolds has also been investigated in several studies. Kon et al. showed that BMSCs can be used to repair bone defects in a sheep model using a porous ceramic scaffold (Kon et al., 2000). After only 2 months, bone formation was enhanced in the BMSC-seeded scaffolds compared with cell-free porous ceramic scaffolds (Kon et al., 2000). Liu et al. showed that in goats with a 26 mm tibia defect, β-tricalcium phosphate (β-TCP; Box 1) scaffolds with osteogenically induced BMSCs can effectively form new bone at 32 weeks (Liu et al., 2010).

**Fig. 2.**
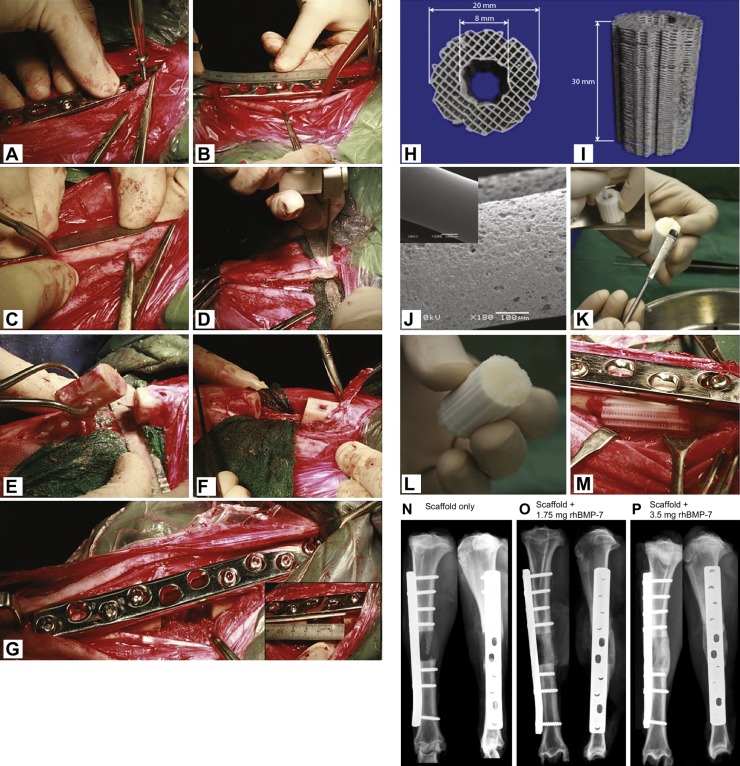
**Surgery and scaffold/rhBMP-7 preparation.** (A-G) Surgical generation of a segmental bone defect and implantation of a TE scaffold. To create a 3 cm segmental tibial defect, the bone was exposed and a dynamic compression plate was temporarily fixed with two screws (A). Subsequently, the screw holes were drilled, the defect middle and osteotomy lines were marked (B,C), and the bone segment was removed after osteotomy (D,E). The periosteum was removed 1 cm on the either end of the tibia defect site before the bone fragments were realigned (F) and fixed with plate and screws (G). (H-M) Top (H) and lateral (I) views of a cylindrical medical grade polycaprolactone tricalcium phosphate (mPCL-TCP) scaffold produced via fused deposition. Prior to transplantation, the scaffolds were surface treated with NaOH to render them more hydrophilic, as demonstrated in the scanning electron microscopy images prior to (J, inset) and after (J) NaOH treatment. To load the scaffolds with the recombinant human bone morphogenic protein BMP-7, the lyophilised BMP-7 was mixed with sterile saline and transferred to the inner duct of the scaffold and onto the contact interfaces between the bone and the scaffold (K,L). The BMP-7-augmented scaffolds were then implanted into the segmental tibial defects (M). (N-P) Representative X-ray images showing segmental tibial defects after 3 months of treatment with the scaffold only (N), the scaffold augmented with 1.75 mg rhBMP-7 (O) or the scaffold augmented with 3.5 mg rhBMP-7 (P), showing superior bone regeneration in the scaffolds with increasing amounts of rhBMP-7 loading due to the potent osteoinductive properties of rhBMP-7. Adapted from Cipitria et al. (2013).

Bone tissue harbours many growth-promoting factors to allow for bone formation, including BMPs, platelet-derived growth factor (PDGF) and insulin-like growth factor (IGF). Addition of growth factors to the scaffolds to enhance bone formation has been assessed in several tibia defect models. Our group investigated the role of recombinant human BMP-7 (rhBMP-7) and composite scaffold composed of medical grade poly-ε-caprolactone (PCL) with β-TCP, in promoting bone regeneration in an ovine CSD tibia defect (Cipitria et al., 2013). We applied 3.5 mg rhBMP-7 to the composite scaffold and observed greater bone formation and superior mechanical properties for the rhBMP-7-loaded composite scaffold compared with the current standard, which is autologous bone grafting, after 12 months (Fig. 2). Niemeyer et al. evaluated the effect of platelet-rich plasma (PRP) growth factors on bone healing. The study compared BMSCs to adipose-derived stem cells (ADSCs) with and without PRP supplementation for 26 weeks. Radiographic evaluation showed that at 10 weeks, the BMSC animals showed more bone formation than the ADSC alone group, but this could be compensated by adding PRP to the ADSCs (Niemeyer et al., 2010).

Another parameter that can affect the quality and quantity of bone healing in the segmental bone defect is the type of fixation device used (Reichert et al., 2009). For clinical translation purposes, it is useful to apply an external fixation device that is used in the clinic and that also supports bone healing. If the fixation is too rigid, it will prevent healing (Perren, 2002). Internal fixation devices, including bone plates and screws or intramedullary nailing, are commonly used to stabilise the tibia defect and mimic the clinical setting (Cipitria et al., 2013; Fernandes et al., 2014; Histing et al., 2016; Pobloth et al., 2017). One group recommended external fixation combined with cylindrical mesh implants instead of internal fixation, as it prevents any interference with the biological responses at the defect site (Gugala et al., 2007). To date, no study has directly compared the effects of different fixation devices on bone healing to determine which is most appropriate for segmental tibia defects.

Additionally, the age of the animal needs to be considered when studying the tibia defect model. Studies have reported tibia segmental defects using both skeletally mature and young sheep. Malhotra et al. demonstrated that the rate of bone growth in femoral and proximal tibia defects of 8, 11 and 14 mm in diameter was higher in skeletally immature (18-month-old) sheep compared with aged, skeletally mature (5-year-old) animals (Malhotra et al., 2014). Reichert et al. advised the use of sheep with an average age of 7-9 years, because secondary osteonal remodelling (Box 1), which would make it more similar in structure to humans, does not take place until sheep reach this age (Reichert et al., 2010a). However, when researchers conduct studies in large animals, younger animals are often preferred due to cost. This can result in an underestimated effectiveness of the TE strategies that are most likely to be applied to the older human population once translated to the clinical setting.

#### Considerations when using the long-bone tibia segmental defect model

One of the biggest challenges in creating an advanced therapy medicinal product (ATMP) is to generate a suitable preclinical model that mimics the real clinical scenario and accurately predicts outcome. To date, variations in the protocols for long-bone tibia segmental defect models, such as the age and sex of the animals, bone stabilisation device, postoperative procedures and appropriate controls, have prevented the development of a standardised large animal long-bone defect model (Cipitria et al., 2013; Fernandes et al., 2014; Pobloth et al., 2017). The long-bone tibia segmental defect model is the most commonly used large animal model to date. However, until a standardised protocol and model can be implemented, thereby eliminating or minimising all the variations listed above, further investigation will be required. The time the animals are followed up after the procedure also varies between studies. Despite reports that a simple fracture in a sheep achieves union in 10-15 weeks (Nakamura et al., 2017), other surgical parameters, such as elevation or reduction of the periosteum at the defect site also influence healing time, and their effects on the local biological healing must be understood in order to standardise the timing of postsurgery follow up (Utvag et al., 1998).

Appropriate controls are another important consideration when evaluating the effectiveness of a TEC in a segmental bone defect model. Some studies use empty defects, whereby the TEC is not implanted, as controls, whilst others do not use any controls at all (Christou et al., 2014). Furthermore, the acceptable bone formation rate in the empty defect, which can be defined as greater than 10% defect area healing throughout the duration of the experiment (Hollinger and Kleinschmidt, 1990), in the control animals is also under debate (Christou et al., 2014) and needs to be standardised. In summary, a consensus to standardise a large animal long segmental bone defect model is required to decrease the gap from bench to bedside for bone TE strategies.

In order to address the limitations in clinical translation of larger animal segmental defect models, the Hutmacher laboratory has standardised and validated a 3 cm and a 6 cm ovine segmental defect model over the past ten years (Berner et al., 2017, 2013; Cipitria et al., 2013, 2015; Reichert et al., 2012a, 2010a, 2011). Different bone tissue engineering concepts were studied in this model over a 3- to 12-month *in vivo* period (Table 4). Consequently, the preclinical testing of a Food and Drug Administration (FDA)-approved and European Conformity (CE)-Marked biodegradable composite scaffold combined with BMP-2 and/or vascularised corticoperiosteal flap (Box 1) did lead to clinical translation in a young patient with a 36 cm tibia defect caused by traumatic injury (Hutmacher, 2017).

**Table 4.**
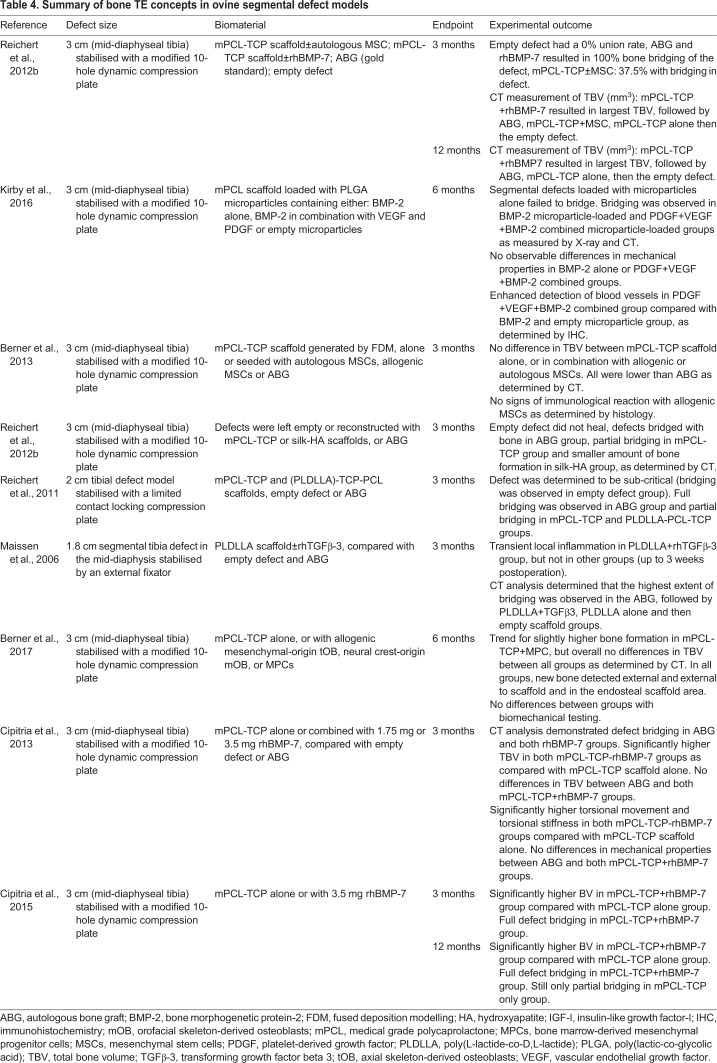
**Summary of bone TE concepts in ovine segmental defect models**

### Comparisons between different bone defect models

As described earlier, most bones arise from the embryonic mesoderm and form via a cartilage intermediate in a process known as endochondral ossification. However, in the calvarial bone, both the neural crest and mesoderm contribute to its development in a process called intramembranous ossification. The differing origin of these bones has important biological consequences. For example, Quarto et al. observed superior osteogenic potential *in vitro* and osseous healing capacities in *in vivo* calvarial defect models in neural crest-derived, as compared to mesoderm-derived, osteoblasts and bone (Quarto et al., 2010). Similarly, neural crest-derived cells were found to produce more mineralised tissue and induce more bone formation than mesoderm-derived cells from long bones (Aghaloo et al., 2010; Reichert et al., 2013). On the other hand, when comparing the rate of regeneration of a tibia burr hole defect (Fig. 1C) with a calvarial defect (Fig. 1A), Lim et al. observed faster healing in the tibia than in the calvarial defect model (Lim et al., 2013). The authors suggested that the differences in healing time between the two defect models could have been caused by increased mechanical loading or the influence of the remaining periosteum at the tibia defect site compared with the calvarial defect site. Clearly, differences in healing capacity exist not only between osteoprogenitor cells (Box 1) of different embryonic origins, but also between different bone defect models. These differences must be considered when analysing the efficacy of TEC on bone regeneration from wounds created at different defect sites.

As described previously, TE techniques for bone regeneration and repair can be applied in animal bone defect models to study new osteoconductive scaffolds and biomaterials. However, the differences in healing capacity between various bone defect sites must be taken into consideration when choosing a model and when comparing with previously published data. These considerations are not only applicable to TE approaches for bone defects induced by trauma, but also to TE approaches used to generate bone to study interactions with cancer cells. In the following section we describe how TE bone is currently being utilised for advanced *in vivo* modelling of primary and secondary bone malignancies.

## Humanised bone approaches for disease modelling

While TE techniques can be applied *in vitro* to study the interaction between bone cells and cancer cells, only *in vivo* models are capable of recapitulating metastatic spread through the host vasculature and subsequent homing to the bone (Dadwal et al., 2016; Hutmacher et al., 2010; Sitarski et al., 2018). Therefore, to understand the importance of the interaction between human bone and human cancer cells in modelling disease pathophysiology, several groups established rudimentary *in vivo* models of a humanised bone environment. In these *in vivo* murine systems, *ex vivo* human bone fragments were ectopically implanted into immunocompromised mice to study human bone physiology in normal and disease states. Fetal bone subcutaneously grafted into SCID mice maintained human haematopoiesis for up to 20 weeks after implantation (Kyoizumi et al., 1992), retained the bone marrow and resident stromal cells, and underwent bone remodelling processes (Nemeth et al., 1999). Conversely, Wagner et al. reported that maintenance of the marrow compartment in subcutaneously implanted adult cancellous bone depended on supplementation with rhBMP-7 at the time of implantation, as the marrow compartment was replaced with fibrous tissue without rhBMP-7 (Wagner et al., 2016). Perhaps this indicates that the fetal bone engrafts in murine models and maintains the marrow compartment and haematopoiesis better than the adult bone, but the availability of fetal bone for research is limited and has important ethical considerations. Human bone graft-bearing mice have also been used to study primary bone malignancies, such as osteosarcoma, and metastatic lesions from prostate and breast cancer, which we discuss in detail below.

Subcutaneous implantation of human bone fragments for studying normal human bone physiology and the role of human bone in disease processes has both benefits and drawbacks. These bone fragments are subject to higher patient-to-patient variability, caused by idiosyncrasies intrinsic to individual patients, and because the procedure itself employs minimal preprocessing steps of the *ex vivo* human tissue. However, this can also be advantageous, as the tissue is minimally handled and therefore representative of the patient's native bone organ. This is important, as the bone stromal and marrow niche is crucial for normal human haematopoiesis and plays a central role in haematopoietic diseases (Kaplan et al., 2005). There are also logistical implications in using bone fragments from surgical waste, as the amount of bone material available for implantation is often not known until after the surgery is performed and the tissue harvested. Furthermore, successful implantation into the mouse is confined to a brief time window following bone tissue collection. Therefore, using human bone fragments from surgical waste does not permit extensive experimental preplanning. Moreover, researchers have reported inconsistencies regarding the maintenance of the endogenous bone marrow, and haematopoiesis, necrosis and infiltration of murine fibrous tissue into the grafted human bone fragment (Holzapfel et al., 2013).

### Tissue engineering techniques to generate the organ bone

**Fig. 3.**
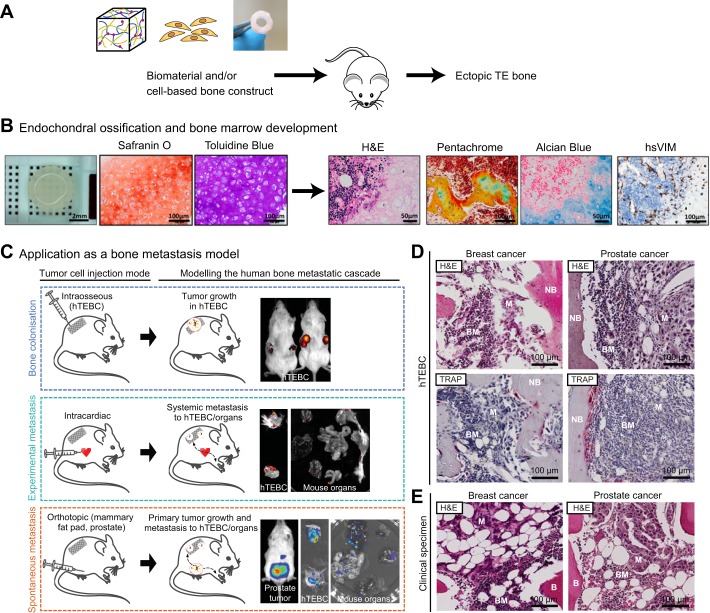
**Tissue-engineered ectopic bone formation for disease model research.** (A) Tissue-engineered (TE) bone construct biomaterials consisting of hydrogel-, cell- or scaffold-based systems can be subcutaneously implanted into immunocompromised mouse models in order to create ectopic, humanised bone in a mouse-as-a-bioreactor-style system. (B) Ectopic bone can form through the process of endochondral ossification, whereby the bone is generated from a cartilage intermediate. Safranin O and Toluidine Blue are histochemical dyes that bind to proteoglycans and glycosaminoglycans and stained the cartilage tissue orange-red and purple, respectively, indicating endochondral ossification in TE bone constructs. Haematoxylin and Eosin (H&E) staining of the TE bone showed marrow infiltration into the bone organ, while pentachrome staining showed black nuclei, yellow bone tissue, green hyaline cartilage, dark red bone marrow and bright red unmineralised osteoid. Alcian Blue staining showed cartilage-associated extracellular matrix in blue and bone marrow in pink. Immunohistochemical staining for human-specific vimentin (hsVIM) demonstrated that the cellular components of the newly formed bone, apart from the bone marrow, were of human origin in cell-based TE bone constructs. (C) The humanised TE bone construct (hTEBC) implanted in a mouse model can be used for disease model research. Cancer cells may be introduced into the mouse system following intraosseous injection to study primary bone tumours and direct cancer-bone interactions, whereas intracardiac injection of cancer cells replicates experimental metastasis in the mouse circulation, allowing investigation of cancer cell homing to distant organ sites. Additionally, cancer cells can be injected at the orthotopic site (e.g. mammary fat pad for breast cancer or intraprostatic injection for prostate cancer studies) in order to study spontaneous metastasis from a primary tumour. (D) Histological examination of metastatic breast and prostate cancer cells (M) in a human TE bone construct with newly formed bone (NB) *in vivo* demonstrated tumour cells residing in the bone marrow (BM) in H&E-stained images. Tartrate-resistant acid phosphatase (TRAP) staining revealed osteoclastic (highlighted by pink staining) breast and prostate cancer metastases in the TE bone. (E) Representative H&E images of patient-derived breast and prostate cancer bone metastases highlight the similarity of the TE bone to the human disease. Adapted from Reinisch et al. (2015) and Martine et al. (2017).

In order to overcome the limitations of native human bone fragments for studying the human bone in normal physiological and disease processes, TE techniques have been employed to form *de novo* bone in *in vivo* models (Fig. 3, Table 5). The major advantage of TE bone is the ability to fully customise the physical properties of the osteoconductive scaffold and the inclusion of osteoinductive growth factors and cell sources. TE bone models can also be used to gain mechanistic insights into bone formation. For example, Eyckmans et al. used an ectopic TE bone model to study bone formation. They delineated the roles of BMP and WNT protein signalling (Box 1) during osteoinduction by knocking down or overexpressing the regulators of these signalling pathways in human periosteal cells and analysing the effects on bone formation (Eyckmans et al., 2010).

**Table 5.**
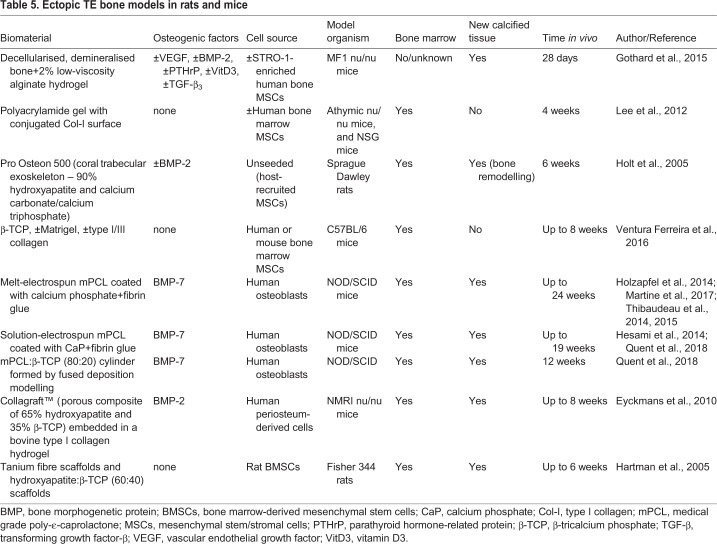
**Ectopic TE bone models in rats and mice**

There are also limitations to TE bone approaches for *in vivo* studies. For example, obtaining sufficient quantities of BMSCs or osteoprogenitor cells of sufficient quality can be difficult, with the source of cells also being an important consideration. Cells derived from fetal sources can be ethically challenging to acquire, and Reinisch et al. suggest that only BMSCs, as opposed to umbilical cord-, skin- or white adipose tissue-derived MSCs, possess the capabilities to form ectopic bone and bone marrow *in vivo* (Reinisch et al., 2015). Furthermore, ectopic bone formation can occur via endochondral ossification (Hartman et al., 2005), a process that seems to be crucial for bone marrow formation in the new engineered organ bone (Eyckmans et al., 2010; Reinisch et al., 2015; Taichman, 2005). Bearing in mind that robust and functional TE bone formation is the critical foundation to study the interactions between human bone and human cancer cells in *in vivo* models, the factors described above need to be carefully considered in order to model disease pathophysiology.

#### Primary bone-related malignancies – leukaemia, multiple myeloma and osteosarcoma

Experimental models of primary bone cancers are generated either by direct injection into the mouse bone marrow, or by intravenous delivery and homing to the bone niche (Cole et al., 2011; Lee et al., 2012). However, they generally perform poorly owing to the relatively low engraftment rate of the cancer cells, and cells from primary bone malignancies that do establish a primary tumour often fail to proliferate or appropriately metastasise (Patel et al., 2012; Sanchez et al., 2009; Sarry et al., 2011). Therefore, in order to better model human primary bone tumours, an approach to humanise the mouse bone and bone marrow has recently been adopted to provide a more relevant and permissible environment for cancer xenografts (Martine et al., 2017; Reinisch et al., 2016).

Leukaemia is a malignant disease in which increased numbers of immature or abnormal leukocytes are produced, leading to the suppression of the normal functioning of the haematopoietic organs, such as red blood cell production from the bone marrow. Recently, Reinisch et al. created an *in vivo* model of normal and malignant human haematopoiesis in which direct injection of patient-derived haematopoietic stem cells (HSCs) and leukaemia cells in the ossicle maintained clonality (Box 1) and enhanced engraftment, demonstrating that a humanised bone microenvironment supports normal and malignant haematopoiesis more effectively than the existing xenotransplantation models (Reinisch et al., 2016). In another study, the TE bone marrow enhanced HSC homing and could support the engraftment and proliferation of the erythroleukaemia (Box 1) cell line TF-1a (Lee et al., 2012).

Multiple myeloma is a haematopoietic malignancy caused by the expansion of bone marrow-resident plasma cells. In one study, multiple myeloma cells were injected into subcutaneous fetal bone grafts in SCID mice. The bone grafts could support metastasis of the myeloma cells from one bone graft to another within the same mouse. Interestingly, injected multiple myeloma cell lines were not detected in the murine bone marrow, indicating that species-specific metastasis of human myeloma cells to human bone occurred (Urashima et al., 1997). No TE bone studies have been performed for multiple myeloma xenograft studies, but based on results from leukaemia studies, this would be a promising avenue of research.

Unlike leukaemia and lymphoma, osteosarcoma is a cancer derived from mesenchymal bone cells and is characterised by osteoblastic differentiation and osteoid formation. In an ectopic bone chip model containing human bone matrix and functional human haematopoietic cells, direct injection of the human primary osteosarcoma cell line SaOS-2 in the ectopically implanted bone resulted in the successful growth of a primary tumour that developed spontaneous lung metastases, a classic metastatic hallmark of the disease (Wagner et al., 2016). To date, no TE bone methods have been applied to study other primary bone malignancies such as chondrosarcoma, Ewing's sarcoma or fibrosarcoma.

Similar bone graft models have also been shown to support species-specific bone metastasis of human prostate cancer (Nemeth et al., 1999; Yonou et al., 2001), as well as breast cancer cells (Kuperwasser et al., 2005).

#### Secondary bone metastases – breast and prostate cancer

Most cancer-related deaths are caused by metastatic spread rather than the primary tumour. Breast, prostate, renal, lung and thyroid cancers preferentially metastasise to the bone (Virk and Lieberman, 2007), and it is postulated that disseminated cancer cells follow the cytokine signalling pathways that are usually used by the HSCs to home to the bone marrow microenvironment (Decker et al., 2016; Reagan and Rosen, 2016). Once there, the disseminated cancer cells lie dormant or interact with the resident bone cells to stimulate growth factor release and other pro-tumorigenic signals (Ottewell, 2016).

In order to determine the factors that influence breast cancer metastasis to the bone, Sieb et al. generated ectopic bone by implanting a silk-based scaffold functionalised with BMP-2 into immunocompromised mice. The authors reported that receptor activator of nuclear factor κB ligand (RANKL; TNFSF11) enhanced breast cancer metastasis to the TE bone and formation of osteolytic lesions (Seib et al., 2015). It is important to note that no human bone-forming cells were included in this model, as the ectopic bone was of mouse origin. Regardless, this study shows an important role of RANKL signalling in breast cancer metastasis to bone, and that TE bone can be used to delineate important molecular mechanisms of disease pathogenesis *in vivo*.

In a humanised system, silk scaffolds seeded with BMSCs supported the metastatic spread of SUM1315 human breast cancer cells from the orthotopic implantation site in the murine mammary fat pad (Moreau et al., 2007). Moreover, a humanised bone model generated by solution-electrospun CaP-PCL scaffolds and human osteoblasts supported the metastatic growth of different human breast cancer cell lines (MDA-MB-231, SUM1315 and MDA-MB-231BO) and osteolytic damage caused by the breast cancer cells could be observed in the TE bone construct (Quent et al., 2018). In the same TE bone model, Hesami et al. described osteoclast-mediated destruction of the bone environment following direct injection of LNCaP and PC3 prostate cancer cells into the TEC (Hesami et al., 2014). Similarly, TE bone formed from melt-electrospun PCL scaffolds seeded with human osteoblasts could create a humanised bone organ in mice (Martine et al., 2017) and could be used to study the homing of breast cancer cells to the humanised bone following intracardiac injection (Thibaudeau et al., 2014). Furthermore, this model was also used to determine the mechanistic insights into the role of integrin β1 in the colonisation of cancer cells on the human bone (Thibaudeau et al., 2015), and to demonstrate species-specific metastasis to the humanised bone following intracardiac injection of PC3 prostate cancer cells (Holzapfel et al., 2014). Although TE bone models have been extensively used for studying breast and prostate cancer metastasis, they haven't yet been applied for renal, lung and thyroid cancers, which also have a propensity to metastasise to the bone.

### Opportunities to improve upon the current TE models in cancer research

All of the TE bone models used for disease research described in this Review are ectopic models; therefore, bone formation relies on an efficient ‘take’ of the scaffold or the *in vitro* TEC by the host model organism. This means that the TE bone construct requires host vascularisation and needs to escape rejection by the host immune system. Furthermore, bone formation at an ectopic site depends on the osteoinductive properties of the engineered scaffold to guide the TE construct towards formation of a functional organ bone. Additionally, TE bone constructs at the ectopic site do not receive the appropriate mechanical stimulation (i.e. loading) to fully function as a mechanically reactive and weight-bearing organ. Owing to their small size and the surgical precision required to create CSDs at sites such as the femur (Box 1) and tibia, implanting a TE bone construct orthotopically would be difficult in mouse models. Using rats for such studies could overcome some of these limitations. Table 6 describes the advantages and disadvantages of using rats and mice for *in vivo* studies.

**Table 6.**
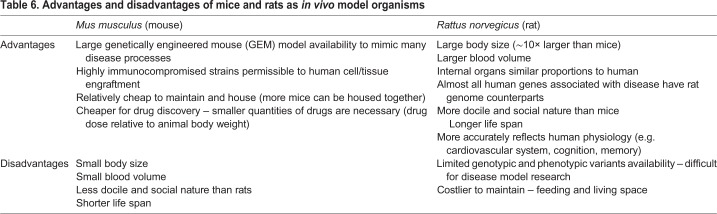
**Advantages and disadvantages of mice and rats as *in vivo* model organisms**

## Conclusion

Exploring new TE approaches to generate new bone for repair or replacement of bone defects in the clinical setting relies on the combination of scaffolds, cells and growth factors. Understanding whether such approaches are suitable and optimised for the translation from bench to bedside requires preclinical testing in animal models. Over recent years, an emphasis has been placed on the optimisation of small and large animal preclinical models of bone loss and regeneration due to the rapidly expanding field of TE. Large animal models offer a suitable system for the testing of TE products used to restore bone defects, whereas small animal models are being explored to model primary and secondary bone-related malignancies. The motivation for the future of preclinical *in vivo* testing must now be to standardise these procedures at every level, from animal species choice to surgical practice. Such standardisation will shrink the gap between the creation of bone TEC to their regulatory approval and clinical testing. This will allow for greater translation of novel experimental TE scaffolds into the clinical practice of restoring traumatic or disease-related bone loss. Wider incorporation of TE bone techniques for disease models will enhance our ability to study the pathogenic interaction between the cancer and the bone, especially for haematopoietic malignancies which have thus far proven particularly difficult to model *in vivo*. Furthermore, humanised TE bone disease models open up opportunities for enhanced therapeutic testing platforms, particularly in the case of human-specific drug treatments or immunotherapies.
